# Web-based rehabilitation after surgical salvage procedures for wrist osteoarthritis: protocol for a randomised controlled trial

**DOI:** 10.1136/bmjopen-2026-127498

**Published:** 2026-11-02

**Authors:** Tobias Tandrup, Maria Wilcke, Elin M Swärd

**Affiliations:** 1 Department of Clinical Science and Education, Södersjukhuset, Karolinska Institutet, Stockholm, Sweden; 2 Department of Hand Surgery, Södersjukhuset, Stockholm, Sweden

**Keywords:** Hand & wrist, Telerehabilitation, REHABILITATION MEDICINE

## Abstract

**Introduction:**

Wrist osteoarthritis (OA) is a disabling condition often affecting individuals of working age and frequently resulting from previous trauma. When non-operative management fails, surgical salvage procedures may reduce pain but require structured postoperative rehabilitation. Telerehabilitation has demonstrated comparable outcomes to standard face-to-face rehabilitation in several musculoskeletal conditions; however, evidence following its effectiveness after wrist OA surgery is lacking. This trial aims to evaluate whether a web-based rehabilitation programme is non-inferior to standard, face-to-face rehabilitation following surgical salvage procedures for wrist OA.

**Methods and analysis:**

This is a prospective, parallel-group, assessor-blinded, randomised controlled non-inferiority trial conducted at a tertiary hand surgery centre. 90 adults undergoing surgical salvage procedures for wrist OA (four-corner fusion, lunocapitate fusion, scaphotrapeziotrapezoid fusion, radioscapholunate fusion or proximal row carpectomy) will be randomised 1:1 to receive either postoperative web-based rehabilitation or standard face-to-face rehabilitation.

The primary outcome is the patient-rated wrist evaluation score measured 12 weeks after immobilisation has ended. Secondary outcomes include pain, health-related quality of life, fear of movement, range of motion, grip strength, patient-reported experience, adherence and cost of rehabilitation.

**Ethics and dissemination:**

Ethical approval has been granted by the Swedish Ethical Review Authority in November 2024. Results will be disseminated through peer-reviewed publications.

**Trial registration number:**

NCT06814821

**STRENGTHS AND LIMITATIONS OF THIS STUDY**

- A randomised, single-blinded, active-controlled design is employed to reduce selection bias.
- Using both objective outcomes and patient-reported outcome measures (PROMs) provides a comprehensive evaluation of treatment effects by capturing physiological changes alongside patients perceived symptoms and functional impact.
- Validated Swedish versions of all PROMs are used. Grip strength and range of motion are measured in a standardised manner according to guidelines developed by the Swedish Quality Registry for Hand Surgery.
- The lack of blinding of participants in this trial may introduce performance bias.

## Introduction

### Background and rationale

 Osteoarthritis (OA) is a chronic disease affecting all joint tissues,^1^ with pain as the primary symptom.^2^ In the hand, OA most commonly affects the interphalangeal joints and the thumb base, whereas wrist involvement is less frequent.^3 4^ Wrist OA is more prevalent in men, tends to present at a younger age^3 4^ and is often caused by past trauma to the wrist.^5^

Non-operative management is first-line, with surgery reserved for cases where exercise therapy, patient education, analgesics, orthoses or corticosteroid injections fail to relieve pain. Numerous studies describe different types of surgical salvage procedures in wrist OA^6–9^ but, to our knowledge, none compare different types of rehabilitation after surgery.

Telerehabilitation for acute and chronic musculoskeletal disorders, including OA, has been used for more than a decade, with demand increasing during and after the COVID-19 pandemic. It is defined as the ‘delivery of therapeutic rehabilitation at a distance using telecommunication technologies’^10^ and encompasses assessments, education and exercise delivered via mobile or tablet applications, websites and related platforms.

Systematic reviews indicate that telerehabilitation is comparable or superior to standard rehabilitation for several musculoskeletal conditions, although the overall evidence quality remains low.^11–14^ Evidence for hand and wrist disorders is limited. In conservatively treated hand OA, operated carpal tunnel syndrome and operatively or non-operatively managed hand and wrist conditions, mobile applications have shown greater improvements in upper-limb function and reduced clinical visits and rehabilitation referrals compared with paper-based home exercise programmes.^15–17^ In surgically treated distal radius fractures, a postoperative digital video programme was similarly effective to traditional therapy in reducing QuickDASH scores at 12 weeks.^18^

Telerehabilitation after OA surgery has demonstrated good patient satisfaction and clinical outcomes, although research has primarily focused on hip and knee OA.^13^ Despite the growing body of literature, no studies have compared web-based or telerehabilitation with standard, face-to-face rehabilitation following surgical salvage procedures for wrist OA.

## Objectives

This trial evaluates whether a web-based rehabilitation programme yields outcomes comparable to those of standard face-to-face rehabilitation following salvage surgery for wrist OA. The primary hypothesis is that web-based rehabilitation is non-inferior to usual care in patient-rated wrist evaluation (PRWE) scores at 12 weeks after immobilisation has ended. Secondary hypotheses are that the intervention group will demonstrate non-inferior outcomes for pain, health-related quality of life, fear of movement, objective function, patient-reported experience, adherence and cost of rehabilitation.

## Methods: patient and public involvement, trial design

### Patient and public involvement

This study is conducted at a regional, highly specialised hand surgery clinic, to which a substantial proportion of patients travel considerable distances to receive care. Most patients undergoing salvage surgery for wrist OA are of working age. Over time, patients have expressed a preference for access to digital rehabilitation options. Although there was no formal patient or public involvement in the design of the study protocol, the development of the web-based platform was informed by feedback from previous patients, whose experiences and preferences were incorporated during the development process.

### Trial design and setting

The trial design is a parallel group, two-arm, pragmatic, randomised, active-controlled, assessor blinded, non-inferiority trial of patients operated with surgical salvage procedures (four corner fusion (4CF), luno-capitate fusion (LC), radioscapholunate fusion (RSL), scaphotrapeziotrapezoid fusion (STT) or proximal row carpectomy (PRC)) for wrist OA. The study adheres to the Standard Protocol Items: Recommendations for Interventional Trials (SPIRIT)^19^ and the results will be reported according to the Consolidated Standards of Reporting Trials (CONSORT) guidelines.^20^ Patients will be randomly assigned (1:1) to receive postoperative web-based rehabilitation or standard face-to-face rehabilitation.

The study is conducted at the Department of Hand Surgery, Södersjukhuset, Stockholm, Sweden.

## Methods: participants, interventions and outcomes

### Recruitment and eligibility criteria

All patients scheduled for surgical salvage procedures due to wrist OA will be screened according to the inclusion and exclusion criteria (table 1). Eligible patients receive digital information about the study and are subsequently contacted by telephone to be invited to participate.

**Table 1:** Inclusion and exclusion criteria

Inclusion
Planned 4CF, LC, STT, RSL or PRC.
Age ≥18 years.
Access to digital identification.
**Exclusion**
Unstable rheumatoid arthritis or other chronic inflammatory arthritis. However, stable inflammatory arthritis with unchanged medication during the last 12 weeks is not an exclusion criterion.
History of gout or pseudogout affecting the hand or wrist.
Symptomatic OA in nearby joints.
Inability to co-operate with the follow-up protocol (language difficulties, severe psychiatric disorder, cognitive impairment, drug addiction, inability to use web-based tools).

4CF, four corner fusion; LC, luno-capitate fusion; OA, osteoarthritis; PRC, proximal row carpectomy; RSL, radioscapholunate fusion; STT, scaphotrapeziotrapezoid fusion.

The first participant was enrolled on 2 April 2025, and recruitment is expected to be completed in 2028.

### Intervention and comparator

#### Treatment given to all participants

Participants from both treatment arms are given oral standard information regarding their operation, immobilisation and rehabilitation by their surgeon when put on the waiting list for surgery.

Participants are operated as inpatients or outpatients depending on the surgeon’s and patients’ preferences. Inpatients from both treatment arms receive written information and a brief oral instruction by a hand therapist regarding maintaining full range of motion (ROM) in non-immobilised joints and reducing oedema. Outpatients from both treatment arms receive written information before discharge from the day care unit.

The timing of rehabilitation depends on the surgical procedure and the preferences of the responsible surgeon. After PRC, wrist exercises begin at cast removal, approximately 4–5 weeks postoperatively. After intracarpal arthrodesis, wrist exercises start once radiological confirmation of fusion has been obtained and the K-wires have been removed. The estimated time to fusion for 4CF, LC, STT and RSL arthrodesis is 8–12 weeks.

Both treatment arms are encouraged to fill out an exercise diary to monitor adherence from week 1–12, after immobilisation ends. A detailed description of the exercise programme is provided in online supplemental file 1.

### Web-based exercise therapy programme (intervention)

The web-based platform is located at the Swedish healthcare system’s national website for information about health and illness (1177.se) and uses a digital communication tool with a secure login procedure. Text, images and videos educate and inform the patient regarding wrist OA pathophysiology, surgical techniques, pain and oedema management and the different rehabilitation phases.

The web-based platform contains the following topics:

- Wrist OA
  - The healthy joint.
  - What is OA? (pathophysiology, epidemiology).
  - What is wrist OA? (wrist anatomy, radiographs).
- Your operation
  - Before your operation (fasting, preoperative hygiene, information regarding the day care unit).
  - Different types of surgery for wrist OA (surgical technique, radiographs, immobilisation).
  - Operation day (images from the operating room, length of operation).
  - Patient-reported outcomes from Swedish Quality Registry for Hand Surgery (HAKIR).
  - Interview with a patient who has undergone surgery.
- After your operation
  - Immobilisation, cast and wrist orthosis.
  - Pain management (medication, weaning schedule).
  - Oedema management (elevation, pumping exercises).
  - Scar management and hypersensitivity.
  - Sick leave.
- Rehabilitation and activities of daily living (ADL)
  - Exercises and ADL during immobilisation (text, images and video).
  - Exercises and ADL after immobilisation, week 1–2 (text, images and video).
  - Exercises and ADL after immobilisation, week 3–7 (text, images and video).
  - Exercises and ADL after immobilisation, week 8–12 (text, images and video).
- Contact
  - Contact your hand therapist (ask questions or request a video consultation).
  - Contact a nurse or your doctor.

At the end of immobilisation participants are sent to the hand therapist on call to get an orthosis and confirm that the participant has logged in to the web platform and understands the online rehab instructions. There are no further scheduled physical consultations, however participants may request additional video consultations and can send text messages to the hand therapist.

### Standard rehabilitation (control group)

The control group receive the same education, exercises and prefabricated wrist orthosis as the intervention group. The information is provided verbally and a booklet with images of the exercises is given. However, they do not get access to the web-based platform and follow-up appointments are face-to-face at the clinic.

At the follow-up appointments, participants perform their exercises and adjustments and corrections are made when needed. The progression of the rehabilitation follows the same timespan as the exercise therapy programme for the intervention group.

The number of rehab sessions per participant varies depending on the needs of the participants and what the treating hand therapist deems necessary. Participants in the control group receive approximately four face-to-face follow-ups with a hand therapist (the average number of postoperative sessions in wrist OA salvage surgery at the department).

All information regarding the exercise programme is documented in the medical record.

### Outcomes

An overview of outcome measures assessment time points is provided in table 2.

**Table 2:** The assessment time points and outcomes collected

Outcome	Preoperatively	End of immobilisation	12 weeks after immobilisation ends
PRWE	X	X	X
Pain NRS	X	X	X
EQ-5D-5L	X	X	X
TSK-17	X	X	X
ROM	X		X
Grip strength	X		X
PREM			X
Adherence			X
Cost (rehabilitation)			X

EQ-5D-5L, five-Level EuroQol five-Dimensional Questionnaire; NRS, Numerical Rating Scale; PREM, patient-reported experience measures; PRWE, patient-rated wrist evaluation; ROM, range of motion; TSK-17, Tampa Scale of Kinesiophobia.

### Primary outcome

The PRWE is a wrist-specific outcome measure consisting of a 15-item questionnaire that assesses pain and functional limitations in daily activities. Scores range from 0 (best) to 100 (worst). PRWE is a well-established instrument with high reliability, validity and responsiveness.^21 22^

### Secondary outcomes

Secondary outcomes include both patient-reported outcome measures (PROM) and objective measurements:

- Pain at rest and during loading, measured using the Pain Numerical Rating Scale (0–10 points).^23^
- Health-related quality of life assessed with the five-Level EuroQol five-Dimensional Questionnaire (EQ-5D-5L) (0–1 points).^24^
- Fear of movement measured using the Tampa Scale of Kinesiophobia (17–68 points).^25^
- ROM, measured with goniometer according to the HAKIR measurement manual.^26^
- Grip strength, measured using a Jamar dynamometer according to the HAKIR measurement manual.^26^
- Patient-reported experience measures.
- Adherence recorded using an exercise diary.
- Cost of rehabilitation, defined as hand therapist-delivered contacts, including face-to-face, telephone and video consultations.

### Adverse events

Participants experiencing symptoms or concerns that could indicate a potential adverse event are instructed to contact their hand therapist or surgeon for assessment. Any adverse events, for example, complex regional pain syndrome, that arise during the study will be recorded and included in the final reporting of outcomes. The exercise programme, whether web-based or face-to-face, is not expected to cause adverse side effects.

### Sample size

This study is designed as a non-inferiority trial with the objective of determining whether web-based rehabilitation yields improvement in PRWE scores that is not clinically inferior to standard rehabilitation. The non-inferiority margin was set to 14 points on the PRWE, corresponding to the minimal clinically important difference (MCID) reported by Sørensen *et al*.^27^ The SD was assumed to be 25 points, based on previous data on patients that have undergone intercarpal fusion.^9^ Using the MCID as the non-inferiority margin is considered appropriate in PROMs,^28^ as differences smaller than the MCID are not perceived as clinically meaningful by patients and therefore represent an acceptable threshold of clinical equivalence.

With 41 patients per group, the trial will have 80% power to demonstrate non-inferiority of web-based rehabilitation, assuming a non-inferiority margin of 14 points, an SD of 25 and a one-sided type I error rate of 5%. To account for potential loss to follow-up and the use of non-parametric analyses, a total of 90 patients will be included.

## Methods: assignment of interventions

### Randomisation and blinding

Randomisation will be performed using a stratified approach, with partial wrist arthrodesis and proximal row carpectomy serving as the stratification criteria. A block size of six is applied to maintain balanced allocation across treatment groups over the course of enrolment. The randomisation sequence is generated using SealedEnvelope.com (Sealed Envelope, London, UK). The sequence is implemented and executed through Research Electronic Data Capture (REDCap) (Vanderbilt University, Nashville, Tennessee, USA), which is used as the operational system for participant allocation.

Randomisation will occur after completion of all baseline assessments to prevent any influence on pre-intervention measurements. Neither patients nor treating hand therapists will be blinded to group allocation due to the practical nature of the interventions. However, the surgeon or hand therapist conducting the 12-week follow-up assessment will remain blinded to treatment allocation to reduce the risk of detection bias.

## Methods: data collection, management and analysis

### Data collection

Baseline characteristics and PROM data are collected electronically through REDCap, with patients accessing the forms by scanning a QR code using their mobile device. Measurements of ROM and grip strength are recorded on paper forms and subsequently digitised in the study database.

Participants may withdraw from the trial at any time. If a participant chooses to withdraw, no further data will be collected, but all data obtained up to the point of withdrawal will be retained for analysis.

### Data management

All study data are stored in REDCap, which is secured by password protection and two-factor authentication, and on a secure server managed by Region Stockholm. Each entry is linked to a participant-specific pseudonym, ensuring that no identifiable information is included in the research dataset. Access to the complete dataset is limited to authorised members of the research team.

### Statistical methods

In accordance with European Medicines Agency guidance on non-inferiority and equivalence trials, analyses must demonstrate robustness and adequately address protocol deviations to preserve assay sensitivity.^29^ The primary statistical analysis will follow the intention-to-treat principle, complemented by per-protocol analyses to ensure a robust evaluation of the study outcomes.^30^ Descriptive statistics for continuous variables will be presented as mean (SD) for normally distributed data and median (IQR) for non-normally distributed data, while categorical variables will be expressed as frequencies and percentages.

To analyse longitudinal differences between the two groups, either a generalised estimating equations approach or a mixed model for repeated measurements will be employed. Baseline values and pain catastrophising, assessed using The Pain Catastrophising Scale,^31^ will be included as covariates in the model. The choice of model will be based on the characteristics of the final dataset, including the structure and mechanism of missing data. If necessary, skewed outcome variables will be transformed using logarithmic or square-root transformations to achieve a more symmetric distribution. The significance level will be set at p=0.05. P values will be reported to three decimal places, except values below 0.001, which will be presented as p<0.001. No adjustments will be made for multiple comparisons. Missing data will not be imputed. No subgroup analyses are planned.

A trial-based cost-utility analysis will be conducted to compare web-based rehabilitation with face-to-face rehabilitation. The primary health outcome will be quality-adjusted life years (QALYs), derived from EQ-5D-5L assessments collected at baseline, at cast removal and 3 months thereafter, with utility scores calculated using Swedish population preference weights. QALYs will be estimated using the area under the curve approach with linear interpolation between time points. Mean utilities and QALYs with 95% CIs will be reported for each group. Baseline EQ-5D-5L will be included as a covariate in all QALY analyses. Resource use will be assessed from the healthcare provider perspective and will include all hand therapist-delivered contacts (in-person, telephone and video). Resource use will be multiplied by unit costs to estimate total costs per participant. Cost-effectiveness will be assessed using the incremental cost-effectiveness ratio, defined as the difference in mean costs divided by the difference in mean QALYs between groups, with differences estimated using regression models including group allocation and adjustment for baseline EQ-5D-5L.

All statistical analyses will be performed using R. The data analyst will be blinded to group allocation to reduce the risk of interpretation bias.

### Monitoring

No independent data monitoring committee will be established for this trial. Given the low-risk nature of the intervention and the absence of planned interim analyses, external monitoring was not considered necessary.

## Ethics and dissemination

The trial received approval from the Swedish Ethical Review Authority in November 2024 (2024-04461-01). The trial was registered on ClinicalTrials.gov in November 2024 under the identifier NCT06814821.

Eligible participants receive written and oral information regarding the study, including potential risks and benefits, before providing written informed consent (online supplemental file 2). Participation is entirely voluntary, and individuals may withdraw from the trial at any time without needing to give a reason for their decision.

Electronic files are protected by password security, and participant information is stored in a pseudonymised format within the study’s electronic database. Any documents containing participant names or personal identification numbers are kept separately from the trial data. All study-related files are retained for 10 years after the completion of the trial.

The findings from the trial will be prepared and submitted for publication in a peer-reviewed medical journal to ensure broad scientific dissemination. De-identified study data may be made available to qualified researchers on reasonable request and subject to approval by the Swedish Ethical Review Authority. Requests should be directed to the corresponding author.

## Discussion

This trial is designed as a non-inferiority study comparing web-based rehabilitation with standard face-to-face rehabilitation following surgical salvage procedures for wrist OA.

The primary aim is to evaluate whether a web-based rehabilitation programme is non-inferior to standard face-to-face rehabilitation in achieving clinical outcomes, while potentially offering advantages in accessibility, resource utilisation and patient autonomy.

The 12-week follow-up was chosen as the primary endpoint because it corresponds to completion of the routine postoperative rehabilitation and follow-up period at our department. As the study focuses on rehabilitation outcomes rather than long-term surgical outcomes, this was considered the most clinically relevant time point.

The randomised design constitutes an important methodological strength. The inclusion of both objective measures and validated PROMs further strengthens the study. This dual-outcome strategy enables a comprehensive evaluation of treatment effects that would not be achievable through objective or subjective measures alone.^32^

Standardisation of measurement procedures and the use of validated PROMs represent another important strength. Grip strength and ROM assessments follow guidelines established by HAKIR,^26^ ensuring consistency and reducing variability in data collection.

Despite these strengths, there are also some limitations to consider. A narrower non-inferiority margin was considered; however, given the large variability in PRWE change scores following salvage surgery for wrist OA,^9^ such a margin would require a prohibitively large sample size and render the study infeasible. The chosen margin therefore represents a clinically justified and methodologically pragmatic balance, ensuring both scientific validity and feasibility.

Blinding of participants is not feasible due to the inherent differences between web-based and face-to-face rehabilitation. The absence of blinding may introduce performance bias, as participants’ expectations or preferences could influence adherence, symptom reporting or perceived benefit. In the standard rehabilitation group, therapists interact directly with participants, which may influence the level of support or guidance provided, whereas no such contact occurs in the web-based group. Although this asymmetry reflects the nature of the interventions, it represents a potential source of performance bias. The study is single-blinded, which may represent a potential limitation to internal validity and should be considered when interpreting the findings. However, blinded assessment of objective functional outcomes reduces the risk of detection bias.

In summary, this non-inferiority trial is methodologically robust, incorporating randomisation, validated outcomes and standardised measurement procedures. While the single-blinding is a recognised limitation, the study is designed to provide clinically meaningful and generalisable evidence on whether web-based rehabilitation can serve as an effective alternative to standard face-to-face rehabilitation. If shown to be non-inferior, web-based rehabilitation could offer patient-centred benefits such as increased flexibility, improved accessibility and reduced demands associated with travel to in-person appointments. Hence, the study addresses important knowledge gaps and contributes novel evidence to inform clinical practice and future rehabilitation strategies in wrist OA.

## Supplementary material

10.1136/bmjopen-2026-127498 online supplemental file 1

10.1136/bmjopen-2026-127498 online supplemental file 2
