# Supplementary material for: Web-based rehabilitation after surgical salvage procedures for wrist osteoarthritis: protocol for a randomised controlled trial
Source: BMJ Open. 2026 Nov 2;16(11):e127498. doi: 10.1136/bmjopen-2026-127498 (PMC13735338; doi:10.1136/bmjopen-2026-127498)
Supplement: Supplementary file 1 [file bmjopen-2026-127498.reviewer_comments.pdf]

PEER REVIEW HISTORY

BMJ Open publishes all reviews undertaken for accepted manuscripts. Reviewers are asked to complete a checklist review form and are provided with free text boxes to elaborate on their assessment. These free text comments are reproduced below.

ARTICLE DETAILS

Title (Provisional)

Web-based rehabilitation after surgical salvage procedures for wrist osteoarthritis: Protocol for a randomized controlled trial

Authors

Tandrup, Tobias; Wilcke, Maria; Swärd, Elin M

VERSION 1 - REVIEW

**Reviewer**	**1**
**Name**	**Yamamoto, Michiro**
**Affiliation**	**Nagoya University Graduate School of Medicine Faculty of Medicine, Department of Hand Surgery**
**Date**	**16-Sep-2026**
**COI**	**None**

Overall Assessment

This is a well-written and methodologically sound study protocol. The authors present a clear and feasible randomized controlled trial evaluating whether web-based rehabilitation is non-inferior to usual care following surgical salvage procedures for wrist osteoarthritis. Given the rapid evolution and widespread adoption of telehealth and web-based communication platforms in recent years, the proposed intervention is highly practical and implementable in contemporary clinical settings. The manuscript is coherent, logically structured, and adheres to established reporting standards for protocol papers.

Major Comments

1. Strength and validity of the primary and secondary hypotheses

The primary hypothesis—that web-based rehabilitation is non-inferior to usual care in PRWE scores at 12 weeks after immobilisation—is reasonable and clinically plausible. Prior evidence from other musculoskeletal and postoperative rehabilitation domains suggests that remote or web-based interventions often achieve comparable outcomes to traditional face-to-face therapy. Therefore, the hypothesis is unlikely to be surprising, but it is nonetheless

important to formally test it in the context of wrist osteoarthritis, where evidence is currently lacking.

The secondary hypotheses (non-inferiority in pain, health-related quality of life, fear of movement, objective functional measures, patient-reported experience, adherence, and cost) are similarly appropriate and aligned with the multidimensional nature of postoperative recovery. These outcomes are well chosen and reflect both patient-centered and clinician-relevant domains.

2. Appropriateness of the included surgical procedures

The inclusion of common salvage procedures for wrist osteoarthritis—four-corner fusion (4CF), luno-capitate fusion (LC), radioscapholunate fusion (RSL), scaphotrapeziotrapezoid fusion (STT), and proximal row carpectomy (PRC)—is justified. These procedures share similar postoperative rehabilitation needs, particularly regarding pain control, edema management, restoration of range of motion, and progressive strengthening. The heterogeneity of surgical techniques is acceptable given the stratified randomization and the pragmatic nature of the trial.

The results of this study may also serve as a foundation for expanding web-based rehabilitation to other hand surgery populations, including distal radius fractures and other traumatic injuries, where remote rehabilitation could be equally beneficial.

3. Feasibility and contemporary relevance

The protocol demonstrates high feasibility. The web-based platform is well integrated into existing healthcare infrastructure, and the intervention is clearly described. Given the increasing acceptance of remote rehabilitation, the study is timely and addresses an important gap in the literature.

4. Potential limitations and impact

While the study is well designed, the expected non-inferiority of web-based rehabilitation may limit the novelty and impact of the findings. The hypothesis is likely to be confirmed, and the results may not dramatically shift clinical paradigms. However, demonstrating non-inferiority still carries meaningful practical implications: if web-based rehabilitation proves comparable to usual care, patients may benefit from increased flexibility, reduced travel burden, and improved access to postoperative therapy.

The authors may consider emphasizing these patient-centered advantages more strongly in the discussion.

Minor Comments

The rationale for selecting PRWE at 12 weeks as the primary endpoint is appropriate, though the authors could briefly discuss whether longer-term outcomes might differ.

Cost-effectiveness analysis at 12 weeks is feasible but represents a short-term perspective; acknowledging this limitation would strengthen the manuscript.

The intervention fidelity and adherence metrics are well described, but additional detail on how therapist–patient communication quality will be monitored could be helpful.

Recommendation

Minor Revision

The protocol is strong overall. Addressing the minor points above would further enhance clarity and contextual relevance.

VERSION 1 - AUTHOR RESPONSE

Reviewer Comments

Major Comments

Answer:

We thank the reviewer for the positive and thoughtful evaluation of our study protocol. We have addressed the specific comments and suggestions below.

Minor Comments

Comment 1:

"The authors may consider emphasizing these patient-centred advantages more strongly in the discussion."

Answer:

Thank you for this suggestion. We agree that the potential patient-centred benefits of web-based rehabilitation are important. We have therefore revised the Discussion section (last paragraph) to further emphasize potential advantages such as increased flexibility, improved accessibility, and reduced demands associated with travel to in-person appointments.

Comment 2:

"The rationale for selecting PRWE at 12 weeks as the primary endpoint is appropriate, though the authors could briefly discuss whether longer-term outcomes might differ.

Answer:

We agree that longer-term outcomes may differ and could provide additional information regarding the persistence of treatment effects. However, the primary aim of this study is to evaluate the effectiveness of the rehabilitation intervention rather than

the long-term outcome of the surgical procedure itself. Therefore, the 12-week endpoint was chosen as it reflects completion of the routine postoperative rehabilitation period and follow-up at our department. This was considered the most clinically relevant time point for evaluating rehabilitation outcomes. We have added a brief clarification regarding this rationale in the Discussion section (3rd paragraph) in the manuscript.

Comment 3:

"Cost-effectiveness analysis at 12 weeks is feasible but represents a short-term perspective; acknowledging this limitation would strengthen the manuscript."

Answer:

The cost outcome is intended to capture rehabilitation-related resource utilization rather than long-term economic consequences of surgery. As the rehabilitation programme is expected to be completed within 12 weeks, we considered this to be the most relevant timeframe for the analysis. Since the scope of this outcome is already specified in the Methods section (Secondary outcomes), no changes to the manuscript were made.

Comment 4:

"The intervention fidelity and adherence metrics are well described, but additional detail on how therapist–patient communication quality will be monitored could be helpful."

Answer:

Thank you for this suggestion. Therapist-patient communication quality will not be formally assessed in the present study. However, participants in the web-based rehabilitation group will have access to the rehabilitation team through secure messaging and video consultations when needed. In addition, patient-reported experience measures are included and may provide indirect information regarding participants' perceptions of communication and support throughout the rehabilitation process. As communication quality is not a primary focus of the current trial, we did not include a dedicated assessment of this aspect, and therefore no changes to the manuscript were made.
