# Supplementary material for: Web-based rehabilitation after surgical salvage procedures for wrist osteoarthritis: protocol for a randomised controlled trial
Source: BMJ Open. 2026 Nov 2;16(11):e127498. doi: 10.1136/bmjopen-2026-127498 (PMC13735338; doi:10.1136/bmjopen-2026-127498)
Supplement: Supplementary file 2 [file bmjopen-16-11-s001.docx]

**Supplementary**

#### Exercise programme

**Exercises and ADL during immobilisation**

- Active unloaded and passive ROM exercises to maintain or regain full ROM in fingers, thumb (if not immobilised), elbow, and shoulder during the immobilisation period. Performed every 2 hours, 10 repetitions.
- Keep their hand elevated at rest and do active pumping exercises to reduce oedema.
- Allowed to use the hand in light ADL e.g., buttoning a shirt, using cutlery, and using a computer. Less than 0.5 kg.

**Exercises and ADL after immobilisation, week 1-2**

- After immobilisation a prefabricated wrist orthosis is used day and night between exercises.
- Exercises focus on active unloaded ROM and gentle passive ROM of the wrist, extension/flexion, radial/ulnar deviation, pronation/supination. Performed 5-6 times a day, for 2 sets of 10 repetitions.
- Allowed to use the hand in light ADL with wrist orthosis e.g., buttoning a shirt, using cutlery, and using a computer. Less than 1kg.

**Exercises and ADL after immobilisation, week 3-7**

- The wrist orthosis is used as protection during loadbearing activities and at risk.
- Progressive active unloaded and passive ROM of the wrist, extension/flexion, radial/ulnar deviation, pronation/supination. Performed 5-6 times a day, for 2 sets of 10 repetitions.
- Isometric strengthening exercises, 10 repetitions, 5-10 seconds, 2 times per day.
- Allowed to use the hand in medium ADL without wrist orthosis e.g., cleaning, and light gardening. Less than 3 kg.

**Exercises and ADL after immobilisation, week 8-12**

- The wrist orthosis is optional, can be used to decrease pain during or after loadbearing activities.
- Continue active and passive ROM exercises.
- Continue isometric strengthening exercises.
- Dynamic strengthening exercises, 10 repetitions, 2 times per day.
- Grip strength, using therapy putty, 10 repetitions, 2 times per day.
- Progressive increase in loadbearing activities. Level of pain determines load.

After 12 weeks participants are encouraged to continue with functional exercises and gradually increased strengthening exercises.
