# Supplementary material for: Web-based rehabilitation after surgical salvage procedures for wrist osteoarthritis: protocol for a randomised controlled trial
Source: BMJ Open. 2026 Nov 2;16(11):e127498. doi: 10.1136/bmjopen-2026-127498 (PMC13735338; doi:10.1136/bmjopen-2026-127498)
Supplement: Supplementary file 3 [file bmjopen-16-11-s002.docx]

**Web-Based Rehabilitation After Surgical Salvage Procedures for Wrist Osteoarthritis**

**Information for Research Participants**

We would like to ask whether you would like to participate in a research study. This document provides information about the study and what participation entails.

**What is the study about and why are you being invited to participate?**

You are being invited to participate because you are a patient at the Hand Surgery Department, have painful wrist osteoarthritis, and are scheduled for surgery involving partial wrist fusion and/or excision of one or more carpal bones.

The aim of the study is to compare the effectiveness of two rehabilitation approaches following surgery:

1. Standard information and rehabilitation with regular in-person visits to our rehabilitation department.
2. Web-based rehabilitation, where instructions and information are provided electronically, while still allowing contact with the rehabilitation team if questions or problems arise.

Following major wrist surgery, rehabilitation is necessary to restore as much mobility, strength, and function as possible. Traditionally, information and exercise instructions have primarily been provided verbally during in-person visits and through printed materials. In recent years, several web-based education and rehabilitation programs have been developed, and scientific studies have shown web-based rehabilitation to be comparable to traditional rehabilitation following hip and knee osteoarthritis surgery. However, the effectiveness of web-based information and exercise programs after wrist osteoarthritis surgery has not previously been evaluated in a scientific study. In this study, we compare a newly developed patient education and exercise program delivered through the 1177 online platform "Support and Treatment" with standard wrist rehabilitation.

The research is conducted by Region Stockholm and Karolinska Institutet. These organizations are responsible for the study. The study has been approved by the Swedish Ethical Review Authority.

**What does participation involve?**

You will be randomly assigned to either standard rehabilitation or web-based rehabilitation after surgery. Treatment will be provided through the Hand Surgery Department at Södersjukhuset or via the department's web application within the 1177 platform.

**Standard Rehabilitation**

Following surgery, you will receive written instructions on how to perform your exercises while your wrist is immobilized in a cast. After cast removal, you will receive an orthosis and meet with a physiotherapist or occupational therapist who will provide written and verbal instructions regarding your home exercise program. You will then attend follow-up visits to monitor progress and adjust the program as needed. Typically, this involves approximately three to four visits, depending on your progress.

**Web-Based Information and Rehabilitation**

Before surgery, you will gain access to an online portal containing comprehensive information about your diagnosis, the surgical procedure, and the rehabilitation process step by step. The portal includes both written information and instructional videos demonstrating the exercises.

When the cast is removed, you will receive an orthosis and meet with a physiotherapist or occupational therapist who will ensure that you know how to log in to the portal and answer any questions you may have. You will then perform the rehabilitation independently but will be able to contact the rehabilitation team by email and schedule video consultations if needed.

**Follow-Up Assessments**

You will meet with a physiotherapist before surgery, at the time of cast removal, and three months after cast removal.

Before these visits, you will complete four questionnaires assessing pain, function, and quality of life, as well as questions about pain and your perception of treatment effectiveness. Each questionnaire takes only a few minutes to complete.

At the first and final follow-up visits, we will also measure wrist strength and range of motion. During rehabilitation, you will additionally complete an exercise diary.

**Possible Risks and Consequences of Participation**

Participation in the study does not involve any additional risks. The same rehabilitation exercises are provided in both the standard and web-based rehabilitation programs. The online portal includes information about expected rehabilitation outcomes and clear instructions on how to contact the rehabilitation team if anything does not progress as expected.

**What Happens to Your Data?**

The study will collect and record information about you. Information regarding wrist function and questionnaire responses will be collected and processed in accordance with the General Data Protection Regulation (GDPR) to prevent unauthorized access.

Information concerning your wrist osteoarthritis, wrist function, and background information such as age, sex, occupation, and similar variables will be stored in your electronic medical record. Selected information will be transferred from the medical record to password-protected research databases for analysis.

Within these databases, your identity will only be represented by a code number, and the stored information cannot be directly linked to you. The code key will be stored in secure files at Södersjukhuset and Karolinska Institutet. Data will be retained for ten years on secure servers at these institutions. Only researchers involved in the study will have access to the research databases. All analyses will be conducted at group level, and you will not be identifiable in any reports or publications resulting from the study.

Region Stockholm is responsible for processing your personal data. Under GDPR, you have the right to access the information held about you free of charge and to request correction of any inaccuracies. You may also request deletion of your personal data or restriction of its processing. However, these rights may be limited when the data are necessary for ongoing research.

To access your data, please contact the principal investigator, Elin Swärd. The Data Protection Officer can be contacted at dataskydd.sodersjukhuset@regionstockholm.se. If you are dissatisfied with how your personal data are handled, you have the right to submit a complaint to the Swedish Authority for Privacy Protection (IMY).

**How Will I Receive Information About the Results?**

You will receive information about your individual assessment results during follow-up visits. The overall results of the study will be published in a scientific journal.

**Voluntary Participation**

Participation is entirely voluntary, and you may withdraw from the study at any time. If you choose not to participate or decide to withdraw, you do not need to provide a reason. Your decision will not affect your future care or treatment.

If you wish to withdraw, please contact the study investigators listed below.

**Study Investigators**

**Tobias Tandrup**, Physiotherapist
PhD student
Email: tobias.tandrup@regionstockholm.se

**Elin Swärd**, Associate Consultant Hand Surgeon
Principal Investigator
Email: elin.sward@regionstockholm.se

**Consent to Participate**

I have received oral and/or written information about the study and have had the opportunity to ask questions. I may keep the written information provided.

- I consent to participate in the study entitled “*Web-Based Rehabilitation After Surgical Salvage Procedures for Wrist Osteoarthritis”.*

| **Place and Date** | **Signature** |
| --- | --- |
|  |  |
|  | **Printed Name** |
|  |  |
